# The cultivation regimes of *Morchella sextelata* trigger shifts in the community assemblage and ecological traits of soil bacteria

**DOI:** 10.3389/fmicb.2023.1257905

**Published:** 2023-09-21

**Authors:** Yan Zhang, Qi Zhao, Stéphane Uroz, Tianpeng Gao, Jing Li, Fengqin He, Rusly Rosazlina, Francis Martin, Lingling Xu

**Affiliations:** ^1^School of Biological Sciences, Universiti Sains Malaysia, Penang, Malaysia; ^2^Xi'an Key Laboratory of Plant Stress Physiology and Ecological Restoration Technology, Key Laboratory of Natural Product Development and Anticancer Innovative Drug Research in Qinling, School of Biological and Environmental Engineering, Xi'an University, Xi'an, Shaanxi, China; ^3^Key Laboratory for Plant Diversity and Biogeography of East Asia, Yunnan Key Laboratory of Fungal Diversity and Green Development, Kunming Institute of Botany, Chinese Academy of Sciences, Kunming, China; ^4^Université de Lorraine, INRAE, UMR Interactions Arbres/Microorganismes, Centre INRAE Grand Est-Nancy, Champenoux, France; ^5^The Engineering Research Center of Mining Pollution Treatment and Ecological Restoration of Gansu Province, Lanzhou City University, Lanzhou, China

**Keywords:** *Morchella sextelata*, community assemblage, bacteriome, continuous cropping, ecological traits

## Abstract

The successful large-scale cultivation of morel mushrooms (*Morchella sextelata*) requires a comprehensive understanding of the soil bacterial communities associated with morel-farming beds, as the interactions between fungi and bacteria play a crucial role in shaping the soil microbiome. In this study, we investigated the temporal distribution and ecological characteristics of soil bacteria associated with morel fruiting bodies at different stages, specifically the conidial and primordial stages, under two cropping regimes, non-continuous cropping (NCC) and continuous cropping (CC). Our findings revealed a significant reduction in the yield of morel primordia during the third year following 2 years of CC (0.29 ± 0.25 primordia/grid), in comparison to the NCC regime (12.39 ± 6.09 primordia/grid). Furthermore, inoculation with morel mycelia had a notable impact on soil bacterial diversity, decreasing it in the NCC regime and increasing the number of generalist bacterial members in the CC regime. The latter regime also led to the accumulation of nutrients in the soil beds, resulting in a shift from a stochastic to a deterministic process in the composition of the bacterial community, which differed from the NCC regime. Additionally, mycelial inoculation had a positive effect on the abundance of potential copiotrophic/denitrifying and N-fixing bacteria while decreasing the abundance of oligotrophic/nitrifying bacteria. Interestingly, this effect was more pronounced in the NCC regime than in the CC regime. These results suggest that the increase in potential copiotrophic/denitrifying and N-fixing bacteria facilitated the decomposition of nutrients in exogenous nutrient bags by morel mushrooms, thereby maintaining nitrogen balance in the soil. Overall, our study provides valuable insights into the interactions between morel mycelia and the associated soil bacteriome as well as the influence of different cultivation regimes on these interactions. These findings contribute to our understanding of the complex dynamics of the soil microbiome and can inform strategies for optimizing morel mushroom cultivation.

## 1. Introduction

Morels (*Morchella* spp.) are highly prized mushrooms known for their exceptional taste and nutritional composition, which includes amino acids, polysaccharides, and trace elements (Liu et al., 2017, 2018; Tietel and Masaphy, 2018). With the growing demand for morel mushrooms that exceeds the availability of wild resources, the cultivation of black morel species (such as *M. sextelata*, *M. importuna*, and *M. exima*) in China has expanded rapidly in recent decades (Zhang et al., 2023a) because of the widespread application of exogenous nutrient bag (ENB) technology, a special type of organic substrate enriched in plant polysaccharides (Tan et al., 2019). Previous studies have demonstrated the presence of complex microbiota associated with morels under natural conditions and production systems (Orlofsky et al., 2021). Changes in the structure and composition of bacterial communities have been observed in the presence of morels and during their development, indicating that morels can modify soil microbiota. These changes seem to coincide with successful morel fruiting, regardless of substrate, cultivation method, greenhouse, or natural habitat (Orlofsky et al., 2021; Tan et al., 2021; Liu et al., 2022; Zhang et al., 2023a). Functional inference analyses have highlighted the important roles of specific bacterial communities associated with nitrogen fixation, nitrification, and nutrient mobilization during the cultivation of morels (Pion et al., 2013; Orlofsky et al., 2021; Yu et al., 2022).

To achieve high yields, it is a common practice to cultivate the same species or isolates of morels continuously on the same farm beds without interruption. However, this intensive cultivation often leads to diminishing yields over time and heightened vulnerability to soil-borne diseases. Similar challenges have been noted in the production systems of other edible fungi, such as *Agaricus bisporus* and *Ganoderma lingzhi*, wherein declines in yield or quality deterioration have been documented (Chen et al., 2015; Yuan et al., 2021). The practice of continuous cropping presents significant hurdles to the advancement of the black morel agroindustry. Previous investigations have revealed that the continuous cultivation of *M. sextelata* is associated with elevated soil nutrient levels, including organic matter, total nitrogen, alkali-hydrolyzable nitrogen, and available phosphorus. This increase in nutrient content is attributed to the decomposition of the raw substrate within ENB. Additionally, imbalances within the soil mycobiome have been observed in systems characterized by continuous cropping (Zhang et al., 2023b).

The patterns of community composition, assembly mechanisms, and ecological functions of soil bacteria, especially within the context of continuous cultivation throughout the entire morel production cycle, remain poorly understood. We postulated that the success or failure of *M. sextelata* establishment and fruiting, alongside the consecutive soil nutrient changes, would influence distinct responses of the soil bacterial community across various developmental stages and cropping regimes. Furthermore, we hypothesized that bacteria exert a pivotal role in facilitating the growth and maturation of morel fruiting bodies. To test these hypotheses, a controlled indoor experiment was set up to investigate the dynamic progression of soil bacterial communities, indicators linked to specific developmental stages and cropping strategies, ecological processes, and functional attributes during morel cultivation under two cropping regimes: non-continuous cropping (NCC) and continuous cropping (CC). Our findings will enhance our understanding of the complex interactions between morels and soil bacteria, the role of the bacterial community in supporting morel growth and development, and contribute to the development of sustainable morel cultivation practices.

## 2. Materials and methods

### 2.1. Experiment description and sampling

**Figure 1:**
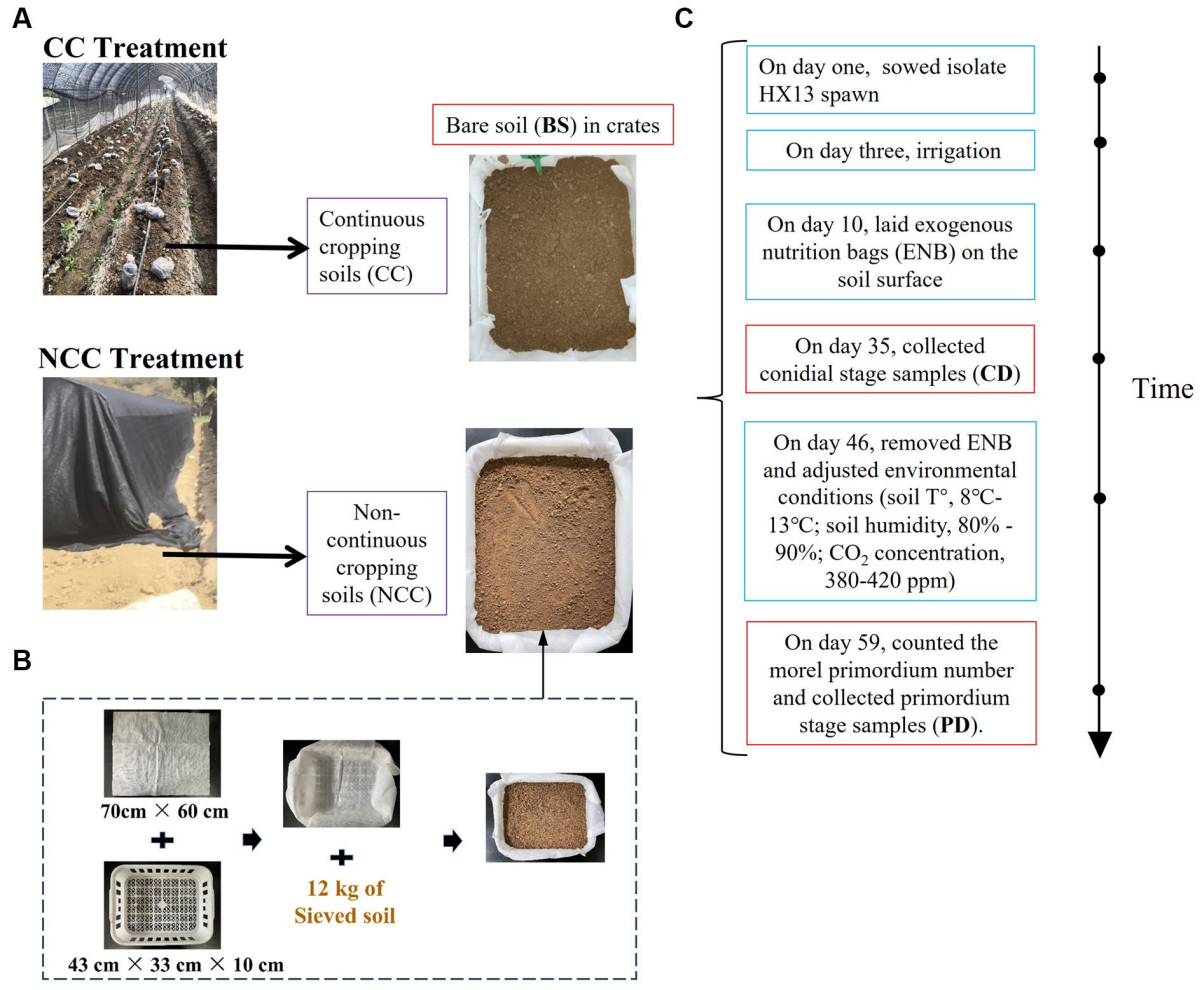
Schematic of experimental design. **(A)** The soil preparation of continuous cropping (CC) and non-continuous cropping (NCC) regimes. **(B)** The crates preparation. **(C)** Cultivation process.

The field experiment was conducted in Hu County, Shaanxi Province, north-western China (34°6′31.05’ N, 108°36′18.04′ E). Black morels (*Morchella sextelata* isolate HX13) were cultivated in a monoculture for two consecutive years (2019–2020). Soils from the NCC and CC regimes were collected, sieved (2 mm), and placed in crates (Figure 1). These crates were then placed in a walk-in chamber at Xi’an University, sown with the HX13 isolate, and managed according to the established morel cultivation procedures. Samples were collected at three stages: bare soil (BS), conidial (CD), and fruiting body primordium (PD). All soil preparation, inoculation procedures, and sample collection procedures were performed as previously described by Zhang et al. (2023b). The primordium yield was estimated by counting the number of morel primordia per 6 × 6 cm grid. For molecular analysis, soil samples were obtained from both NCC and CC regimes. Four replicates were collected for each stage (BS, CD, and PD), resulting in 24 samples. Soil cores, with a diameter of 2.5 cm and spanning the full thickness of the soil, were collected from each crate and mixed thoroughly. Subsequently, 15 g of the mixed soil was used for further DNA extraction and related analyses. All soil samples were immediately frozen in liquid N and stored at −80°C until further processing. Soil physicochemical characteristics, including soil pH, organic matter content, total N, alkali-hydro N, total phosphorus, available phosphorus, and total potassium, were measured according to previously established protocols (Zhang et al., 2023b).

### 2.2. DNA extraction, PCR amplification, and sequencing

Soil DNA was extracted using the TIANamp Soil DNA Kit (TIANGEN, Beijing, China). For amplification of 16S rDNA, specific primers targeting the V4 hypervariable regions, namely 515F-806R primers (5′- GTGCCAGCMGCCGCGGTAA-3′ and 5’-GGACTACHV GGGTWTCTAAT-3′), were used (Edwards et al., 2015). The PCR products were purified using a Qiagen Gel Extraction Kit (QIAGEN, Hilden, Germany) and subjected to paired-end sequencing on an Illumina NovaSeq sequencer at Novogene Biotechnology Co., Ltd (Tianjin, China). Sequencing data were processed using the QIIME pipeline software (Caporaso et al., 2010). Bacterial sequences were trimmed and assigned to respective samples based on their barcodes. Subsequently, cluster analysis was performed to group the sequences into Operational Taxonomic Units (OTUs) at a 97% identity threshold (Blaxter et al., 2005). Representative sequence alignments for each OTU were generated using MUSCLE (Edgar, 2004). Taxonomic assignment of representative sequences was performed using the SILVA 132 SSU database (Quast et al., 2012). Relative abundance data for taxa were generated by scaling the read count for each taxon across samples using the total-sum scaling method. Archaea, chloroplasts, and mitochondrial 16S rRNA genes were excluded from further analysis. Raw sequencing data were deposited in the NCBI Sequence Read Archive (SRA) database under the accession number PRJNA993383.

### 2.3. Statistical analysis

Statistical analyses were performed using R v4.2.2 (R Core Team, 2021) and SPSS version 23.0 software (SPSS Inc., Chicago, IL). Chao1 (Chao, 1984) and phylogenetic diversity (Faith, 1992) indices were used to assess differences in alpha diversity at the OTU level between the three stages in the NCC and CC regimes, providing comprehensive measures of species richness and evolutionary distance. Bacterial beta diversity was visualized using principal coordinate analysis (PCoA) plots (Minchin, 1987) and evaluated by permutational multivariate analysis of variance (PERMANOVA), using the adonis function of the vegan package (Oksanen et al., 2013). Community-level niche breadth, which indicates habitat specialization, was calculated using the spaa package (Zhang et al., 2016). The distributions of generalist, specialist, and neutral taxa were determined using the EcolUtils R package (Salazar, 2018). To quantify the contributions of different ecological processes to the microbial community structure and succession in different treatments, a null-modeling-based statistical framework (Zhou and Ning, 2017) was employed. The beta nearest taxon index (βNTI) was calculated using 999 randomizations. A value of |βNTI| ≥ 2 indicates dominant deterministic processes, whereas a |βNTI| < 2 indicates dominant stochastic processes (Liu et al., 2022). Based on both βNTI and Bray-Curtis-based Raup-Crick Index (RCBray) values, deterministic and stochastic processes were classified into five ecological processes: heterogeneous selection (βNTI< −2), homogeneous selection (βNTI> +2), dispersal limitation (|βNTI| < 2 and RCBray>0.95), homogenizing dispersal (|βNTI| < 2 and RCBray< − 0.95), and ecological drift (|βNTI| < 2 and |RCBray| < 0.95) (Xiong et al., 2021b). Indicator OTUs, identified through indicator species analysis and likelihood ratio tests (LRT) (Hartman et al., 2018), were determined based on collective abundances exceeding 0.03. The correlations between bacterial communities, soil physicochemical variables, relative abundance, and primordium yield in NCC and CC soils were explored using Mantel tests (Xiong et al., 2021a), and taxonomic differences in indicator OTUs among different regimes and stages were evaluated using stacked bar charts and heatmaps and analyzed using the R package dplyr (Yarberry and Yarberry, 2021; Xiong et al., 2021b). Potential trophic behavior (i.e., oligotrophs vs. copiotrophs) and roles in the N cycle (i.e., nitrifying, denitrifying, and N fixation) were established based on published studies. Data are presented as mean ± SEM, with the number of samples (n) indicated in each group. Differences between samples were analyzed using one-way analysis of variance (ANOVA) with Duncan’s multiple range test and an independent sample t-test. Results were considered significant at *p* < 0.05.

## 3. Results

### 3.1. Impact of the cultivation procedure on primordium yields, soil morel mycelium abundance, and soil properties

**Figure 2:**
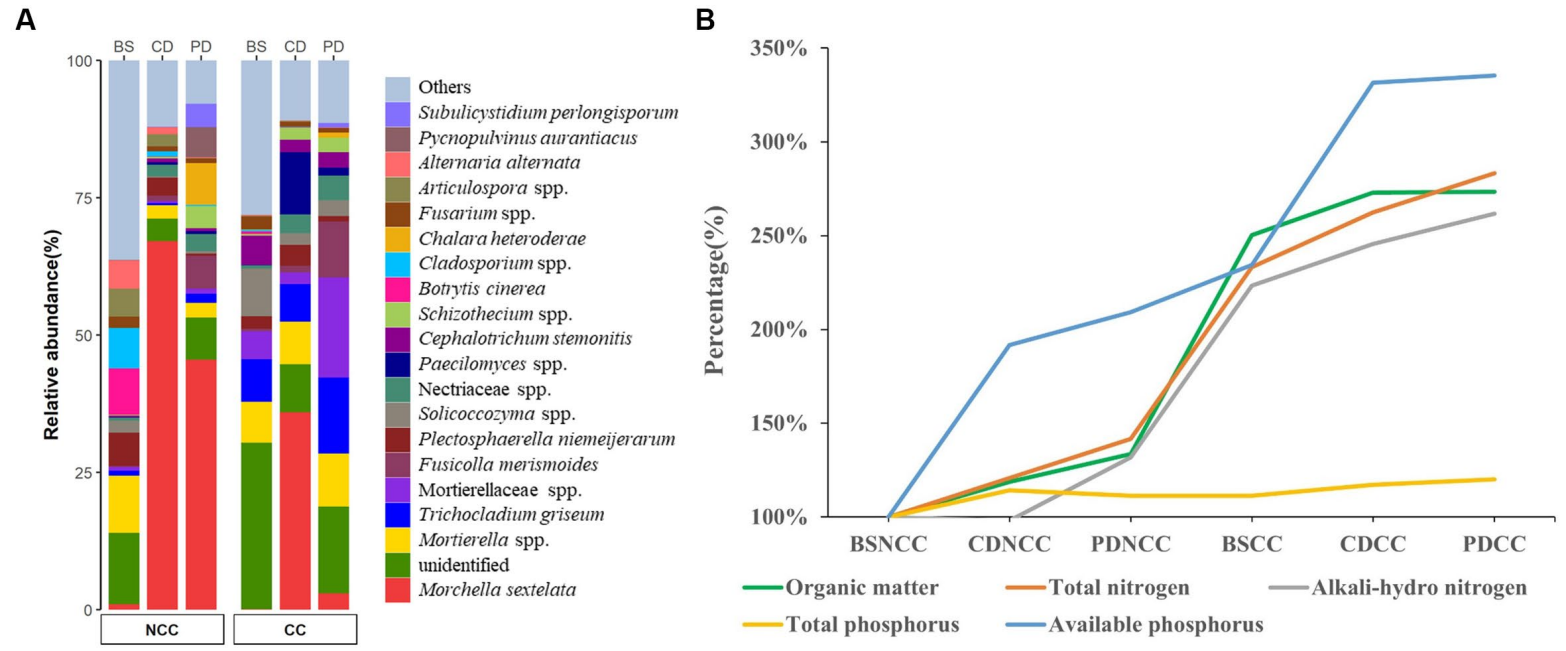
Soil morel mycelium abundance and soil properties. **(A)** Relative abundance (%) of *M. sextelata* and other main taxa/genera detected at different stages [i.e., bare soil (BS), conidial (CD), and primordial (PD)] under both non-continuous cropping (NCC) and continuous cropping (CC). **(B)** Ratio line chart of soil physicochemical characteristics over time.

The primordium yields were significantly higher in the NCC regime, with an average of 12.39 ± 6.09 per grid, compared to the CC regime, where the yield per grid was much lower at 0.29 ± 0.25 per grid. This stark contrast in primordium production corresponds to a significant reduction of 99.7% in the CC regime compared to the NCC regime. rDNA metabarcoding analyses also revealed a notable decrease in the relative abundance of *M. sextelata* in the soil under the CC regime compared to that under the NCC regime, further supporting the decline in morel abundance under continuous cropping (Figure 2A). Furthermore, the CC regime of *M. sextelata* had a profound effect on the soil properties, particularly in terms of nutrient content. The content of organic matter, total N, alkali-hydrolyzable N, and available P increased throughout the course of morel cultivation (Figure 2B and Supplementary Table S1). This suggests that the successive cultivation of *M. sextelata* has a significant influence on soil properties and specifically affects its nutritive content.

### 3.2. Impact of cropping regime on the diversity, community composition and structure of the bacterial communities across the three developmental stages of *Morchella sextelata*

**Figure 3:**
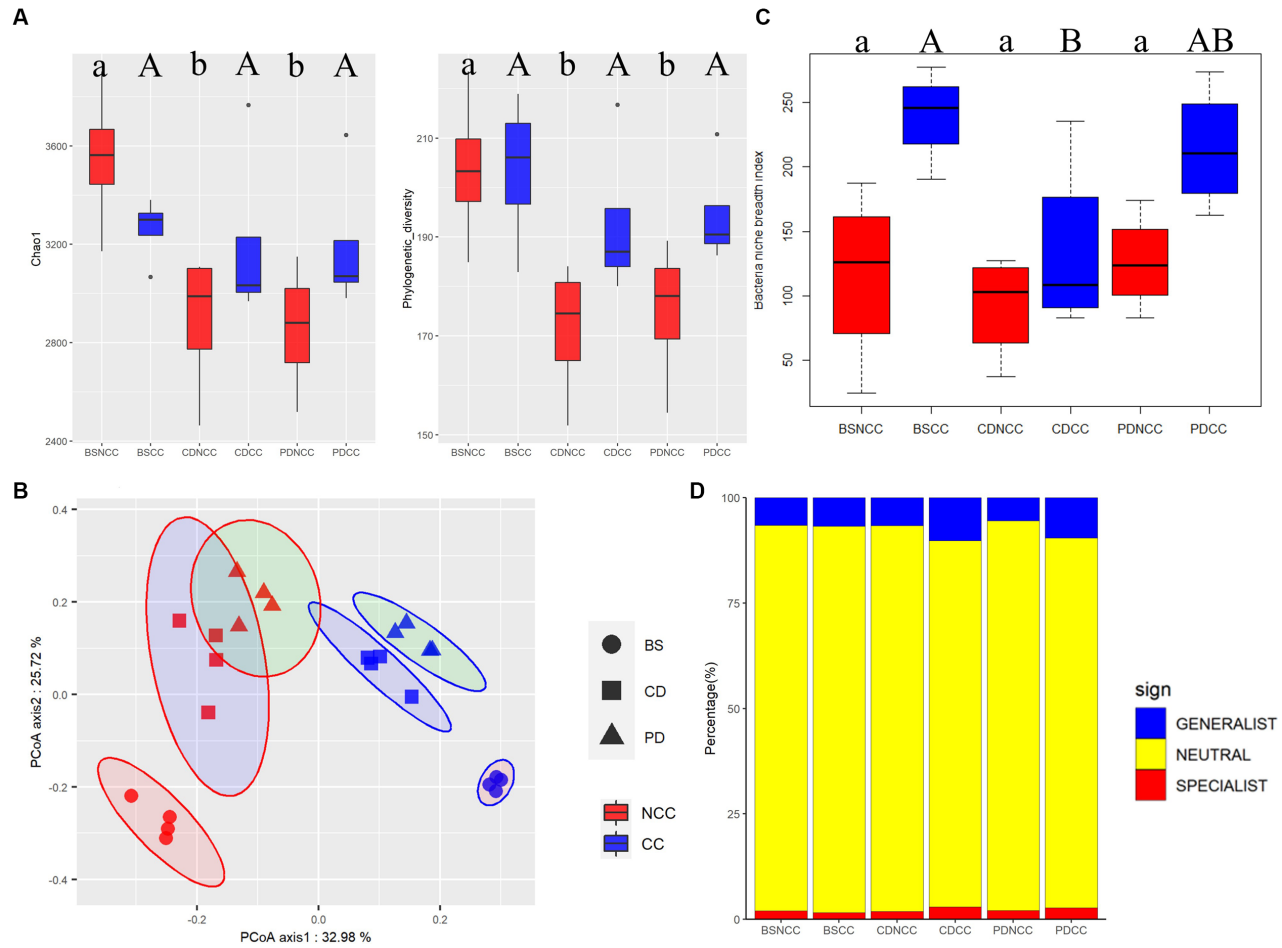
Temporal dynamics of diversity and distribution patterns of crop-associated bacteriomes under the NCC and CC regimes at stages BS, CD, and PD. **(A)** Diversity of the bacterial community of *Morchella sextelata*, as indicated by Chao1 and Faith’s phylogenetic diversity. All values are presented as mean ± SE. Different lowercase letters indicate (*p* < 0.05, Duncan’s test). **(B)** Principal coordinate analysis of the soil bacterial community of *M. sextelata* based on the ray-distance metric. The percentage value for each axis represents the proportion of the total variation explained. The samples collected from NCC and CC are shown in red and blue, respectively. Cycles, squares, and triangles represent the bare soil (BS), conidial stage (CD), and primordial stage (PD), respectively. **(C)** Box plots showing the mean niche breadth of the soil bacterial communities. Lower letters represent *p* < 0.05; **(D)** Relative contributions of habitat generalists and specialists in soil bacterial communities.

After removing contaminating plant and mitochondrial sequences, the remaining high-quality sequences used for analysis totaled 1,558,026 (Supplementary Table S2), corresponding to 9,155 bacterial OTUs. Alpha diversity analysis revealed significant decreases (*p* < 0.05) in both Chao1 and Faith phylogenetic diversity indices under the NCC regime after morel spawn inoculation at the CD and PD stages (Figure 3A and Supplementary Table S3). The highest values of Chao1 (3549.50 ± 299.18) and Faith’s Phylogenetic diversity (203.73 ± 15.83) were observed in the pre-inoculation baseline samples of the NCC regime (BS_NCC). However, at the CD and PD stages, both Chao1 (Chao1CD_NCC: 2887.25 ± 301.79 and Chao1PD_NCC: 2857.75 ± 270.60) and Faith’s Phylogenetic diversity (FaithCD_NCC: 171.26 ± 14.31 and FaithPD_NCC: 174.97 ± 14.96) decreased and fell within a similar range of values. In contrast, no significant differences were observed in Chao1 and Faith’s phylogenetic diversity indices under the CC regime at any of the developmental stages (BS, CD, and PD) (Figure 3A).

Potential variations in the bacterial community composition associated with different cropping regimes and stages were assessed using PERMANOVA and PCoA (Figure 3B). These analyses revealed that the structure of the bacterial community was primarily influenced by the developmental stage (*R*^2^ = 30.5%, *p* < 0.001), followed by the cropping regime (*R*^2^ = 29.4%, *p* < 0.001). The niche breadth of the soil bacterial community was the highest in the BS samples of the CC regime (Figure 3C). Following morel spawn inoculation, niche breadth exhibited a decreasing trend in the CC regime, whereas no significant differences were observed among the three developmental stages in the NCC regime (Figure 3C). Furthermore, the proportion of both generalist and specialist bacterial taxa increased in the CC regime at the CD and PD stages, with generalists showing a greater increase than at the BS stage (Figure 3D).

Taxonomic composition analysis showed that the dominant bacterial taxa belonged to the phyla Proteobacteria, Bacteroidetes, and Acidobacteria (Supplementary Figure S1), and the families Comamonadaceae, Flavobacteriaceae, and Oxalobacteraceae (Supplementary Figure S2). Notably, the relative abundance of all three dominant families was higher in the NCC regime than in the CC regime.

### 3.3. Evidence of the processes explaining the bacterial community assembly in each cropping regime

**Figure 4:**
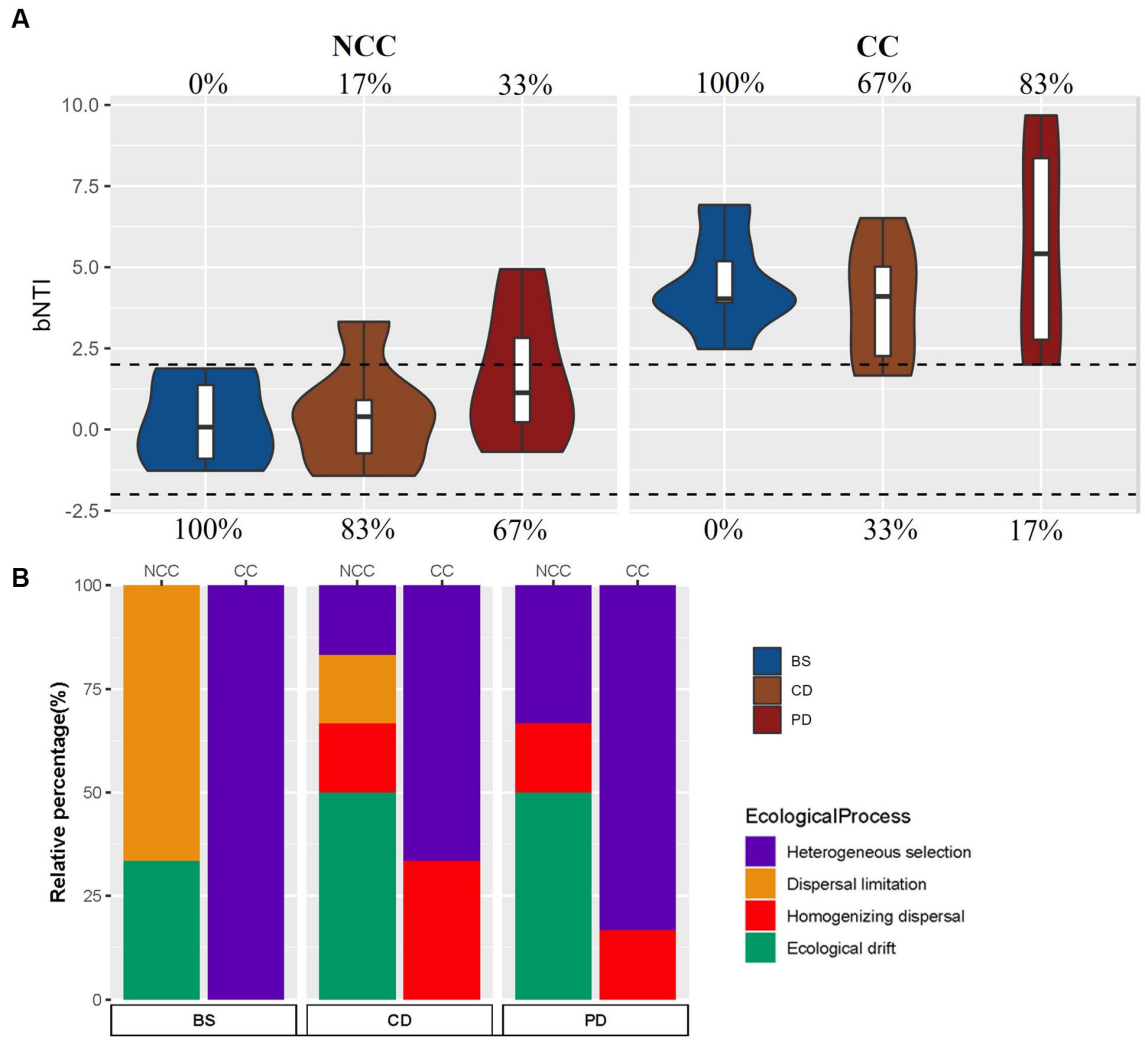
Relative influence of the ecological assembly process in the NCC and CC cropping systems. **(A)** βNTI values for different communities and cropping systems. The percentages above and below the violin plot represent the relative contributions of deterministic and stochastic processes, respectively, and **(B)** the assembly processes of different communities and cropping systems using the null model.

To gain insights into the ecological processes driving the structure of bacterial communities, we performed a βNTI analysis. The results indicated that in the NCC regime, stochastic processes played a prominent role in the structure of the soil bacterial community (Figure 4A). The assembly of the bacterial community in the BS stage was mainly influenced by dispersal limitation, whereas ecological drift was the predominant process in the CD and PD stages (Figure 4B). In contrast, deterministic processes significantly contributed to the assembly of the bacterial community in the CC regime. Heterogeneous selection emerged as the main ecological process shaping the bacterial community assembly (Figure 4B). A comparison of the βNTI patterns revealed how the cropping regime influenced the ecological processes governing bacterial community assembly, with CC leading to a shift from stochasticity to determinism.

### 3.4. Soil properties as deterministic factors explaining the change of bacterial community

**Figure 5:**
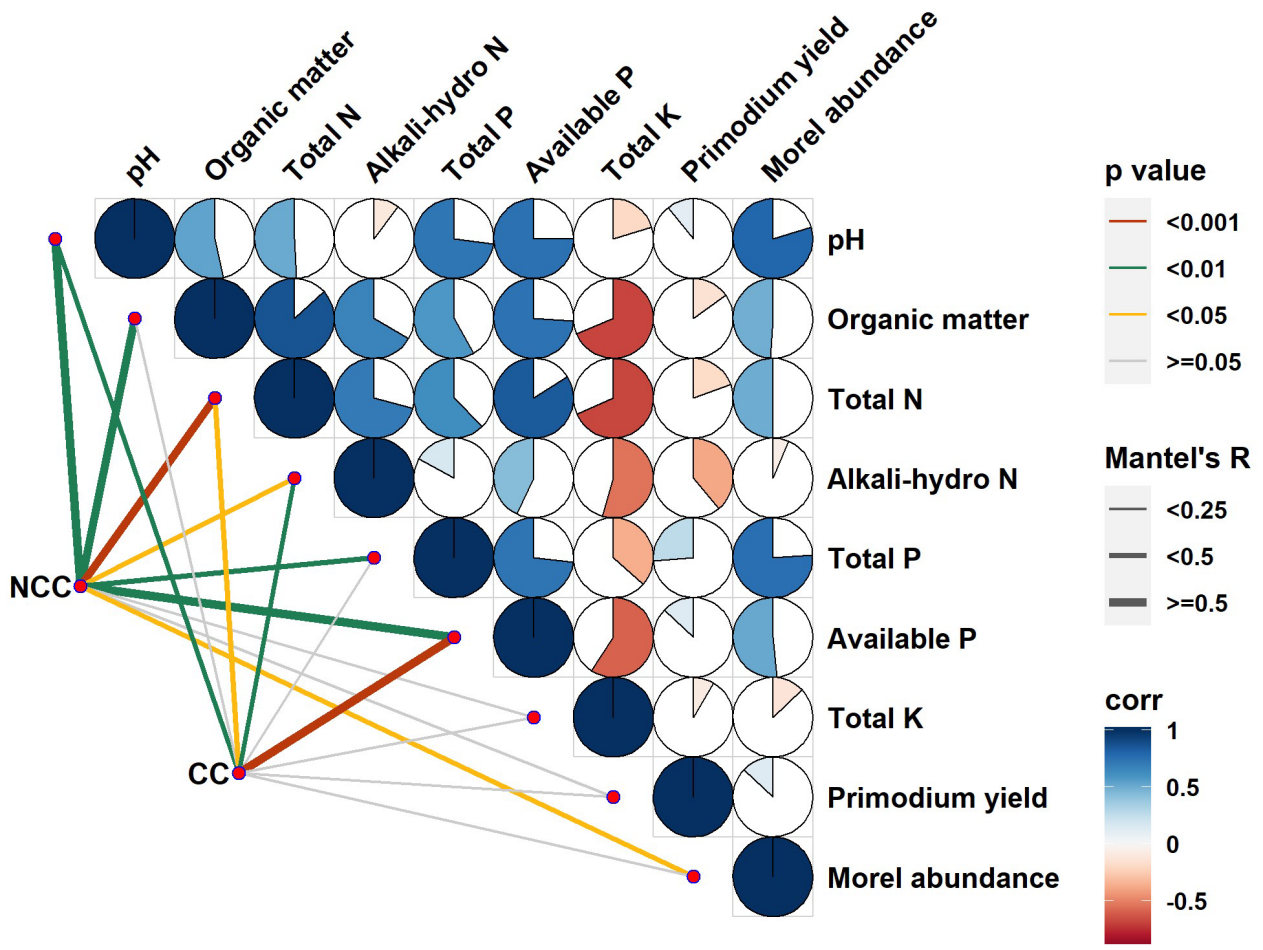
Correlations between bacterial communities and soil physicochemical characteristics, relative abundance of morel soil, and primordial yield in the NCC and CC regimes. Different bacterial communities (in the NCC and CC regimes) were related to each physicochemical variable, the relative abundance of morel soil, and primordial yield by Mantel tests. The proportion of pie indicates correlation strength, with a higher proportion representing a higher correlation strength.

To examine the relationships between bacterial community composition and various factors, such as soil properties, relative abundance of morels, and yield of morel primordia, we conducted a Mantel test (Figure 5 and Table 1). Stronger correlations were observed in the NCC soils compared to the CC soils between the bacterial community and soil properties such as pH, organic matter, total N, and total P. Specifically, organic matter and total P were significantly correlated with bacterial community composition but only in the NCC regime. Conversely, alkali-hydro N and available P content in the CC soils exhibited higher R values and more significant differences in *p* values compared with the NCC soils. Regarding the potential association between morel abundance and yield, strong correlations were found between the bacterial community composition and morel abundance under the NCC regime. However, no correlation was observed in the CC regime. These results suggest that the successful establishment of *M. sextelata* in the soil affects the bacterial community. No relationship was found between the bacterial community composition and morel primordium yield in either the NCC or CC regimes.

**Table 1:** Spearman’s correlations between bacterial community and soil physicochemical characteristics, morel abundance, and yield based on Mantel tests.

Variables	NCC		CC	
	*R* value	*P* value	*R* value	*P* value
pH	0.7307	<0.01	0.4593	<0.01
Organic matter	0.5439	<0.01	0.2036	> = 0.05
Total nitrogen	0.7069	<0.001	0.3463	<0.05
Alkali-hydro nitrogen	0.2654	<0.05	0.4060	<0.01
Total phosphorus	0.4619	<0.01	0.1942	> = 0.05
Available phosphorus	0.7980	<0.01	0.8467	<0.001
Total potassium	0.0393	> = 0.05	0.0316	> = 0.05
Morel abundance	0.4481	<0.01	0.1302	> = 0.05
Primordium yield	0.0805	> = 0.05	−0.0410	> = 0.05

NCC, non-continuous cropping regime; CC, continuous cropping regime.

### 3.5. Identification of indicators of stage of developments and of production

**Figure 6:**
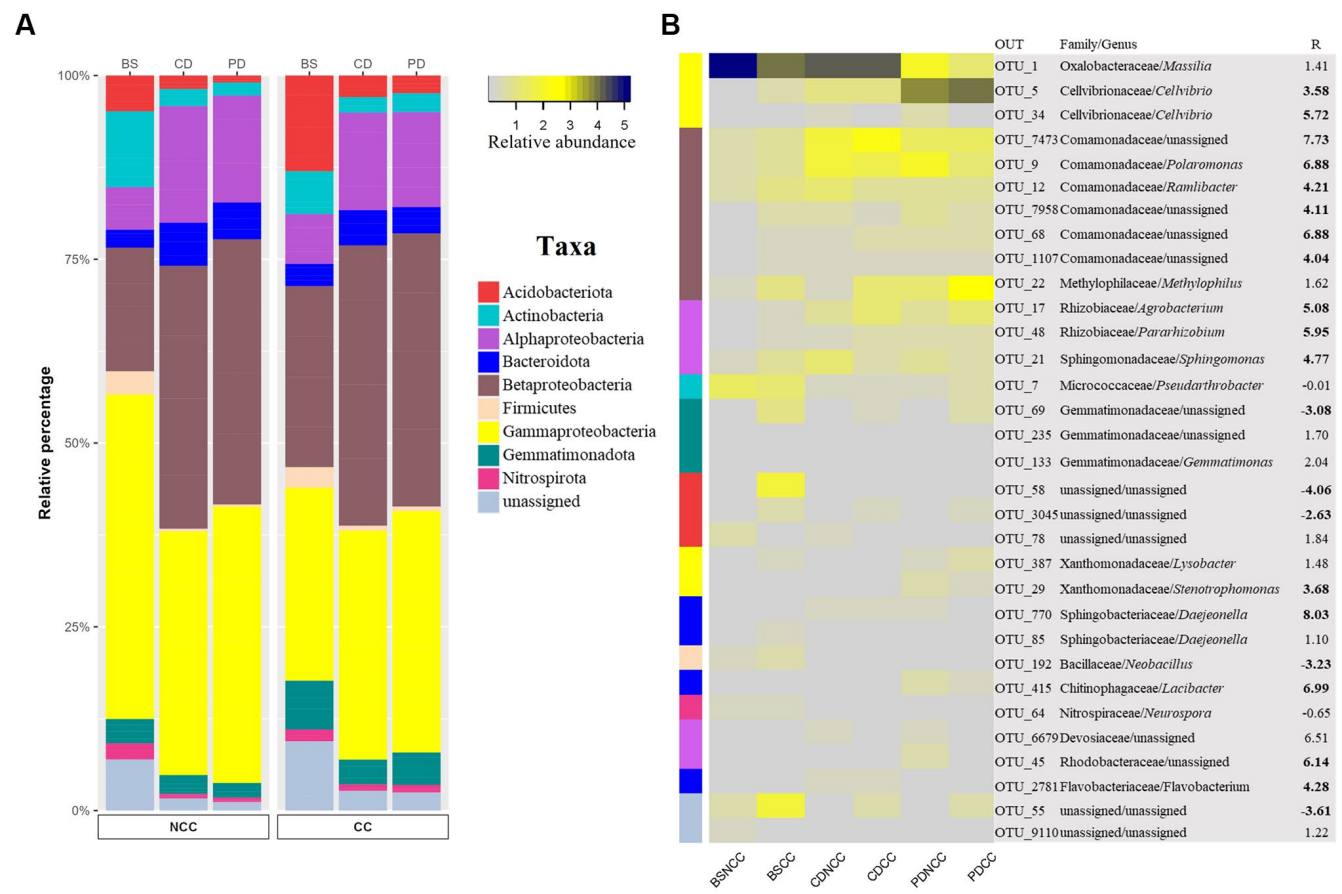
Taxonomic composition of bacterial indicator OTUs present at >3% relative abundance in all treatments. **(A)** Stacked bar chart of the relative percentages of the abundant indicator OTUs at the phylum level (Proteobacteria at the class level). **(B)** Heatmap of bacterial abundance indicator OTUs for each treatment. Colors in the left column indicate the same taxa as A. The numbers on the right represent the *R* value of Spearman’s correlation between the relative abundance of each bacterial family and the abundance of Morel fungi in the soil. Significant correlation coefficients are noted in bold font (*p* < 0.05).

To identify specific bacterial OTUs that exhibited varying abundances across cropping regimes and developmental stages, we conducted indicator species analysis (Hartman et al., 2018). This analysis identified 1,216 indicator OTUs (*p* < 0.05), with indicator OTUs having a relative abundance exceeding 3% considered as abundant indicator OTUs (Figure 6 and Supplementary Table S4). These abundant indicator OTUs were affiliated with nine phyla (at the class level for Proteobacteria): Acidobacteria, Actinobacteria, Alphaproteobacteria, Bacteroidetes, Betaproteobacteria, Firmicutes, Gammaproteobacteria, Gemmatimonadota, and Nitrospirota (Figure 6).

Spearman’s correlation analysis was performed to examine the correlation between the abundance of the indicator OTUs and *M. sextelata* across all stages in the NCC and CC regimes. The results revealed that all abundant indicator OTUs belonging to Alphaproteobacteria, Bacteroidetes, Betaproteobacteria, and Gammaproteobacteria exhibited a positive relationship with morel abundance in the soil (Supplementary Table S5). In contrast, Acidobacteria, Actinobacteria, Firmicutes, Nitrospirota, and Gemmatimonadota were negatively correlated with Morel abundance (Supplementary Table S5).

In terms of lifestyle, the taxa positively associated with morel abundance were mainly classified as potential copiotrophic bacteria (Goldfarb et al., 2011; Spring et al., 2015; Bennett et al., 2020). Conversely, taxa negatively related to morel abundance correspond to potential oligotrophic bacteria (Bledsoe et al., 2020; Lu et al., 2022).

**Figure 7:**
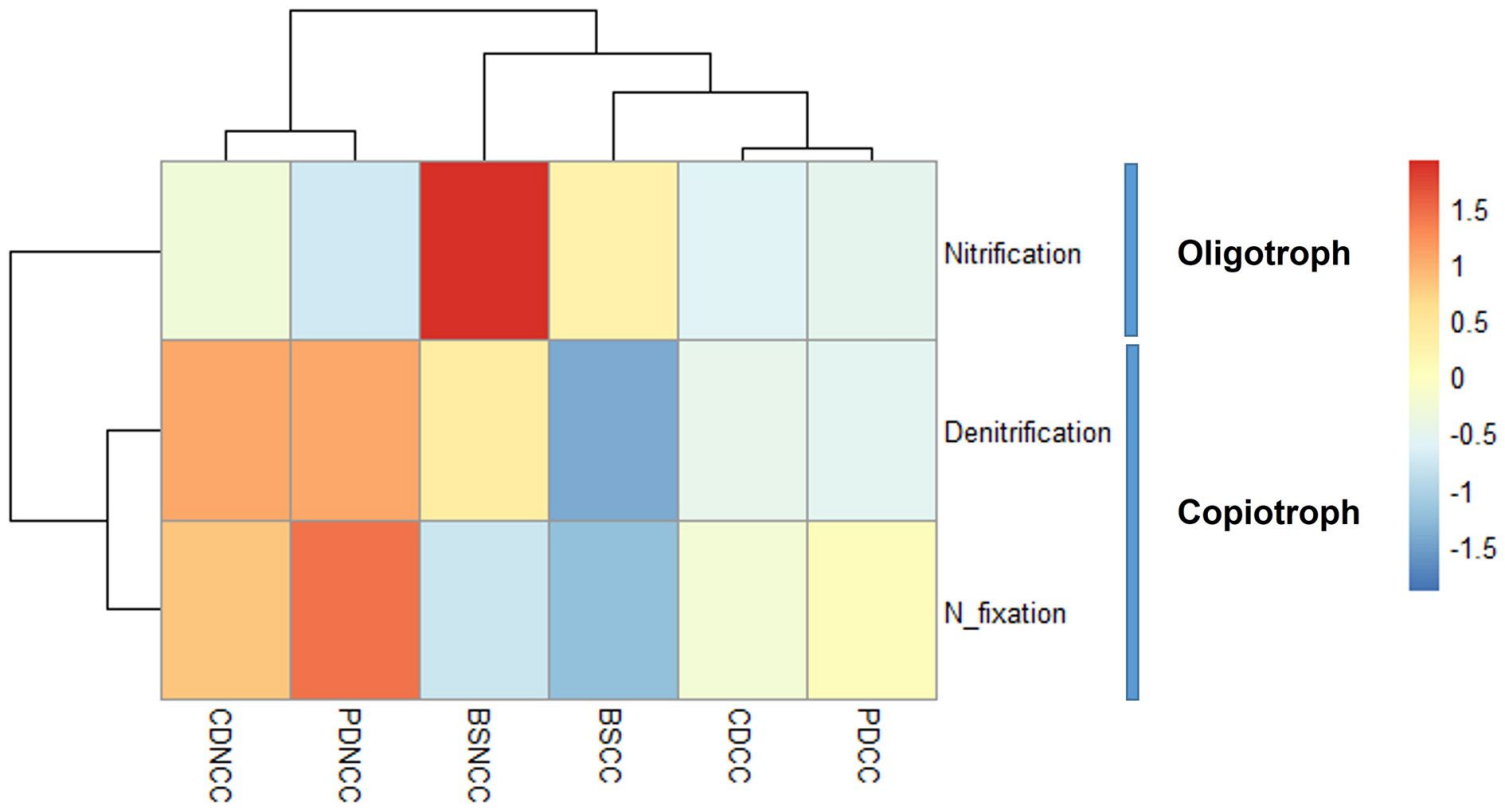
Heapmap of trophic types and *N*-cycling functions of the abundant indicator OTUs.

Among the taxa that were positively correlated with morel abundance, several families have been identified as N-fixing bacteria, including Rhizobiaceae, Devosiaceae, Rhodobacteraceae, and Xanthomonadaceae (Rivas et al., 2002; Altshuler et al., 2019; Rosselli et al., 2021). In addition, families such as Cellvibrionacea, Comamonadaceae, Sphingomonadaceae, Methylophilaceae, Sphingobacteriaceae, and Flavobacteriaceae have been identified as denitrifying bacteria (Khan et al., 2002; Li et al., 2019; Zhou et al., 2019, 2021; Vishnyakova et al., 2022). In contrast, taxa that showed a negative correlation with morel abundance, including Actinobacteria, Firmicutes, Gemmatimonadota, and Nitrospirota, at the phylum level, were identified as potential nitrifying bacteria (Zhou et al., 2020; Lu et al., 2022). Notably, one specific OTU associated with the phylum Actinobacteria (OTU 7) exhibited both nitrifying and denitrifying capabilities (Wendeborn, 2020). Furthermore, following the inoculation of *M. sextelata* in the soil under both NCC and CC regimes, the population of oligotrophic/nitrifying bacteria decreased, whereas copiotrophic/denitrifying and nitrogen-fixing bacteria showed a more pronounced increase in the NCC regime (Figure 7).

## 4. Discussion

### 4.1. Underlying mechanisms of bacterial community assembly under NCC and CC cropping regime

The assembly of bacterial communities in morel cultivation under different cropping regimes, namely NCC and CC, displayed distinct patterns of stochasticity and determinism across the BS, CD, and PD stages. Stochastic processes, primarily driven by dispersal limitations and ecological drift, played a prominent role in the NCC regime, particularly in the BS stage, whereas deterministic processes, dominated by heterogeneous selection, were prevalent throughout all stages in the CC regime. The prevalence of stochasticity at the BS stage in the NCC regime was expected because of the prior state of the soil as an untilled wild field with a low nutrient content. Previous studies have indicated that stochastic processes are more pronounced in wild bacterial communities due to limited nutrient availability, which hampers microbial dispersal (Wang et al., 2013; Yu et al., 2021). Furthermore, the introduction of morel spawns in the NCC regime can be seen as an invasion of a highly abundant alien species, resulting in the rapid and widespread growth of *M. sextelata* and intense competition with other soil microbes. This competitive asymmetry accounts for the significant reduction in bacterial diversity within the soil communities. Because competitive asymmetries often lead to smaller species populations, ecological drift has become a more influential factor in shaping communities (Gilbert and Levine, 2017). Consequently, random ecological drift likely governed the community shifts after spawn inoculation at the CD and PD stages in the NCC regime.

In contrast, deterministic processes played a prominent role in shaping bacterial community composition at all stages of the CC regime. This shift from stochasticity in the NCC regime to determinism in the CC regime can be attributed to the changes in nutritional balance in the soil after the CC regime because the expansion of the morel exhausts some specific nutrients, resulting in the accumulation of unutilized nutrients, which confers a selective advantage to certain bacterial taxa that are better adapted to new nutritional conditions. Heterogeneous selection, driven by specific environmental conditions and nutrient gradients in the CC regime, has become the primary ecological process influencing bacterial community assembly.

These findings indicate that the choice of cropping regime has a significant impact on the ecological processes governing the bacterial community dynamics during morel cultivation. The transition from stochasticity to determinism in the CC regime underscores the importance of managing nutrient levels and understanding the ecological interactions between morels and soil bacteria to implement sustainable cultivation practices. Moreover, these findings contribute to a broader understanding of the underlying mechanisms governing the bacterial community assembly in agricultural systems.

In addition to the impact of mycelial proliferation and invasion, the cultivation of *M. sextelata* resulted in the enrichment of many fungal decomposers (Zhang et al., 2023a), further leading to an increase in soil organic matter (SOM), total N, alkali-hydrolyzable N, and available P content under the NCC regime. This increase was attributed to the decomposition of ENB, which is a specialized culture substrate enriched in plant polysaccharides, by *M. sextelata* and other fungal decomposers present in soil beds (Zhang et al., 2023b). Fungal decomposers decompose complex organic matter, such as the substrate in ENB, into simpler compounds, releasing essential nutrients, such as nitrogen, phosphorus, and carbon back into the soil. This is the basic function of saprophytic fungi as decomposers in the ecosystem. Experienced morel growers measure the N and P content in the soil before inoculation with spawn because of the adverse effects of high soil nutrition on the differentiation of morels (Zhang et al., 2023a). Changes in soil nutrient concentrations can significantly influence the selective environment and drive turnover in bacterial community composition across different successional stages (Jorgensen et al., 2012). In cases where the initial SOM levels are low or there is a substantial increase in SOM due to nutrient addition, elevated nutrient content can induce divergence in community composition through variable selection, known as a heterogeneous process (Feng et al., 2018). Therefore, in the NCC regime, deterministic heterogeneous selection was evident in the CD and PD stages, although it was not the dominant process. In contrast, in the CC regime, the deterministic process was strongest at the BS stage because of the nutrient increase resulting from two years of continuous cropping of *M. sextelata.*

However, after two years of successful fruiting production, imbalances emerged in the CC regime, resulting in a significant decline in *M. sextelata* primordium formation. Soil colonization by *M. sextelata* mycelia was unsuccessful, as indicated by low soil morel abundance and poor fruiting body primordium production. Although deterministic processes remained dominant in the CD and PD stages in the CC regime, a stochastic process of homogenizing dispersal emerged. This process involves a high rate of dispersal among communities, which results in similar community structures (Stegen et al., 2012). The decreasing trend in niche breadth after morel inoculation in the CC regime further supported this finding. The dispersed bacterial community displayed generalist characteristics and was capable of utilizing a wide range of soil resources and habitats. This broad habitat colonization ability facilitates effective stochastic dispersion (Xu et al., 2020) and narrows niche breadth (Zhang et al., 2018). Overall, our results underscore the intricate interplay between stochastic and deterministic processes in shaping the assembly of bacterial communities during morel cultivation under different cropping regimes. The NCC regime exhibited a stronger influence of stochastic processes driven by initial soil conditions and morel inoculation. In contrast, the CC regime demonstrated a predominance of deterministic processes, particularly heterogeneous selection at earlier stages and the emergence of homogeneous dispersal at later stages. These findings enhance our understanding of the ecological mechanisms underlying bacterial community dynamics and succession in morel cultivation, emphasizing the significance of both deterministic and stochastic processes in shaping microbial communities in agricultural systems.

### 4.2. Trophic types of abundant indicator OTUs

Our findings revealed a distinct difference between the characteristics of bacterial OTUs positively associated with morel abundance and those negatively associated with morel abundance. Morel-abundance-positive OTUs were predominantly composed of copiotrophic bacteria, which are known for their rapid growth and reproduction in nutrient-rich environments. These bacteria have high metabolic rates and rely on a continuous supply of organic matter for their growth. Copiotrophic bacteria are commonly found in environments with a high organic matter content, such as recently fertilized soils (Leff et al., 2015). In contrast, the majority of morel-abundance-negative-related OTUs were oligotrophic bacteria. Oligotrophic bacteria have adapted to survive in nutrient-poor environments. They have slower growth rates and lower metabolic rates than copiotrophic bacteria. However, oligotrophic bacteria are efficient at extracting nutrients from their surroundings, allowing them to thrive under nutrient-limited conditions (Leff et al., 2015).

The enrichment of copiotrophic bacteria in morel-abundance-positive OTUs aligned with the increase in soil nutrients following morel inoculation. Copiotrophs thrive in nutrient-enriched environments (Goldfarb et al., 2011; Leff et al., 2015). However, it is worth noting that in the CC regime, despite the higher SOM content compared to the NCC regime, the abundance of copiotrophic bacteria was lower. Furthermore, no significant correlation was found between SOM content and community patterns in the CC regime. These findings suggest that while soil nutrient levels may contribute to the enrichment of copiotrophic taxa, the establishment of morel mycelial networks plays a more crucial role. It is plausible that the presence of morels and their associated activities, rather than nutrient levels alone, shaped the abundance patterns of the copiotrophic bacteria. This indicated that morel establishment influences the ecological function of copiotrophic bacteria during morel cultivation. The mechanisms underlying the relationship between the microbes and copiotrophic bacteria require further investigation. Our results highlighted a clear distinction between morel-abundance-positive copiotrophic bacteria and morel-abundance-negative oligotrophic bacteria. These findings enhance our understanding of microbial dynamics and functional interactions in morel cultivation, and provide insights into the role of copiotrophic bacteria and their ecological functions in this agricultural system.

### 4.3. Bacterial-morel interaction and N metabolism

The family Pseudomonadaceae, particularly *Pseudomonas* spp., is particularly important for morel cultivation. Although no OTUs belonging to the Pseudomonadaceae family were identified as abundant indicator OTUs, they were ranked among the top 10 abundant families and showed a positive relationship with the abundance of *M. sextelata* mycelia in the soil. Previous studies have emphasized the significance of *Pseudomonas putida*, a member of the Pseudomonadaceae family, as a bacterium farmed by the saprotrophic and ectomycorrhizal soil fungus *M. crassipes* (Pion et al., 2013). Similar associations between *Pseudomonas* spp. and *M. sextelata* fruiting have also been observed in outdoor greenhouses (Benucci et al., 2019). *Pseudomonas* has also been found to be accompanied by the induction of fruiting body formation and an increased yield of fruiting bodies during cultivation of other mushrooms, such as *Agaricus bisporus* and *Pleurotus ostreatus* (Cho et al., 2008; Vieira and Pecchia, 2022). These findings underscore the widespread occurrence of bacterial-fungal interactions (BFI) in various ecological contexts (Steffan et al., 2020).

Denitrifying bacteria are responsible for the conversion of nitrate to N_2_ (Vishnyakova et al., 2022). This denitrification process is closely linked to SOM, because heterotrophic bacteria utilize organic carbon sources for their metabolism during denitrification (Burford and Bremner, 1975). For example, the Comamonadaceae family is known for its ability to perform heterotrophic denitrification when supplied with organic compounds as electron donors (Khan et al., 2002). This finding aligns with the significant correlation observed between the community patterns in the NCC regime and SOM content. The reduced organic N content likely acts as a key factor in triggering bacterial farming by *M. crassipes* (Pion et al., 2013; Lohberger et al., 2019).

## 5. Conclusion

**Figure 8:**
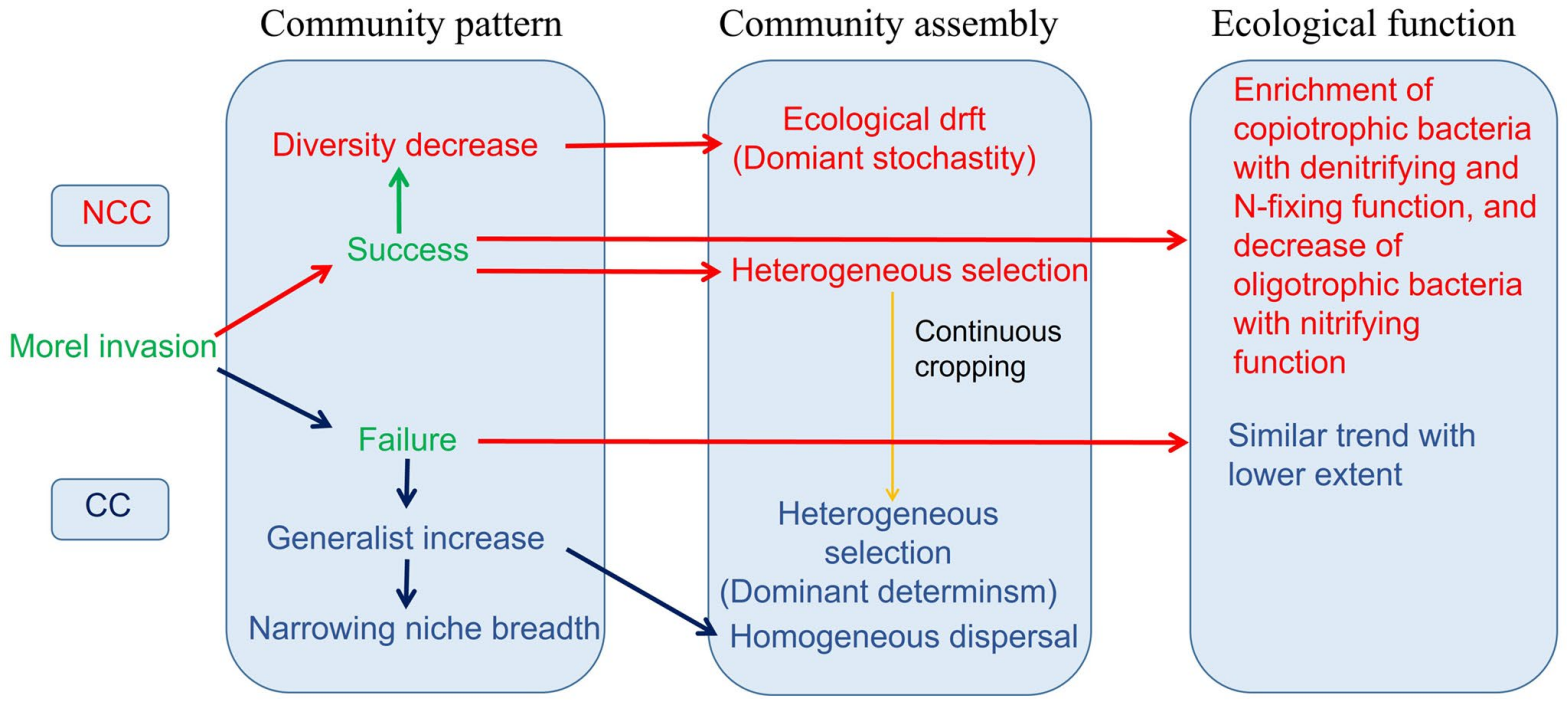
Responses of bacterial community pattern, assembly, and ecological function in NCC and CC regimes during morel cultivation.

This study revealed distinct responses of the bacterial community in terms of community patterns, assembly processes, and ecological functions under NCC and CC regimes (Figure 8). The successful establishment of morels in the soil resulted in a decrease in bacterial diversity, whereas failure of morel invasion in the CC regime led to the dispersal of generalist bacterial members and a reduction in niche breadth. Continuous cultivation of *M. sextelata* influences the assembly of the bacterial community, shifting it from a stochastic process to a deterministic regime. Furthermore, the inoculation of morels had a more pronounced effect on enriching copiotrophic bacteria with denitrifying and N-fixing functions while simultaneously reducing the abundance of oligotrophic bacteria with nitrifying functions. This effect was particularly evident in the NCC regime compared to the CC regime. These findings provide valuable insights into the potential use of bacteria in soil improvement strategies to address challenges associated with continuous cropping in the agricultural industry.

## Data availability statement

The datasets presented in this study can be found in online repositories. The names of the repository/repositories and accession number(s) can be found at: https://www.ncbi.nlm.nih.gov/bioproject/PRJNA993383.

## Author contributions

YZ: Conceptualization, Data curation, Formal analysis, Investigation, Methodology, Project administration, Resources, Software, Writing – original draft, Writing – review & editing. QZ: Data curation, Formal analysis, Investigation, Methodology, Writing – original draft. SU: Supervision, Writing – review & editing. TG: Methodology, Formal analysis, Funding acquisition, Writing – original draft. JL: Data curation, Investigation, Writing – original draft. FH: Data curation, Formal analysis, Funding acquisition. RR: Formal analysis, Methodology, Writing – original draft, Writing – review & editing. FM: Funding acquisition, Project administration, Supervision, Writing – review & editing, Investigation, Writing – original draft. LX: Data curation, Formal analysis, Funding acquisition, Investigation, Supervision, Writing – original draft, Writing – review & editing, Methodology, Project administration, Resources, Software, Validation.

## Funding

The author(s) declare financial support was received for the research, authorship, and/or publication of this article. This research was funded by the National Natural Science Foundation of China (32270530) and the Special Plan of the Education Department of the Shaanxi Provincial Government (Grant No. 21JC028), the Key Research and Development Project of the Department of Science and Technology of Shaanxi Province (Grant No. 2021NY-068), the Innovation Foundation of Science and Technology Bureau of Xi’an (Grant Nos. 2020KJWL05 and 2020KJWL21), and Science and Technology Project of the Science and Technology Bureau of Xi’an (Grant Nos. 20193016YF004NS004 and 21NYYF0023), and the French National Research Agency (ANR) as part of the “Initiative for the Future’ program (ANR-11-LABX-0002-01, Lab of Excellence ARBRE).

## Conflict of interest

The authors declare that the research was conducted in the absence of any commercial or financial relationships that could be construed as a potential conflict of interest.

## Publisher’s note

All claims expressed in this article are solely those of the authors and do not necessarily represent those of their affiliated organizations, or those of the publisher, the editors and the reviewers. Any product that may be evaluated in this article, or claim that may be made by its manufacturer, is not guaranteed or endorsed by the publisher.
