# Supplementary material for: The cultivation regimes of Morchella sextelata trigger shifts in the community assemblage and ecological traits of soil bacteria
Source: Front Microbiol. 2023 Sep 21;14:1257905. doi: 10.3389/fmicb.2023.1257905 (PMC10552182; doi:10.3389/fmicb.2023.1257905)
Supplement: Supplementary file 1 [file Data_Sheet_1.pdf]

Supplementary Material

The cultivation of Morchella sextelata triggers shifts in the community assemblage and ecological traits of soil bacteria

Yan Zhang [1,2], Qi Zhao[3], Stéphane Uroz[4], Tianpeng Gao[2,5], Jing Li[2], Fengqin He[2], Rusly Rosazlina [1,*], Francis Martin[4,*], Lingling Xu [2,4,*]

*** Correspondence:** Rusly Rosazlina: rosazlinarusly@usm.my

Francis Martin : francis.martin@inrae.fr

Lingling Xu: missilexll@163.com

1 Supplementary Figures and Tables

1.1 Supplementary Figures

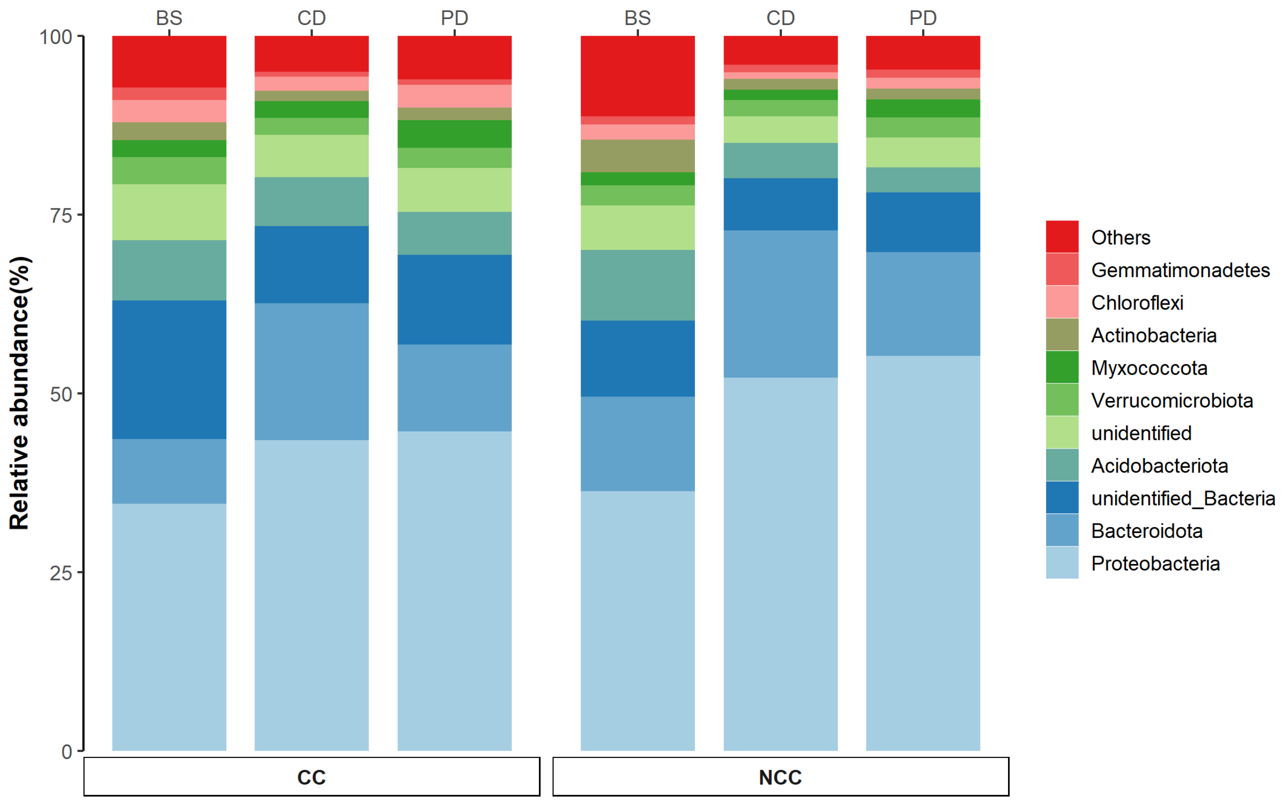

Supplementary Figure 1. Top 10 relative abundances of bacteria at the phylum level.

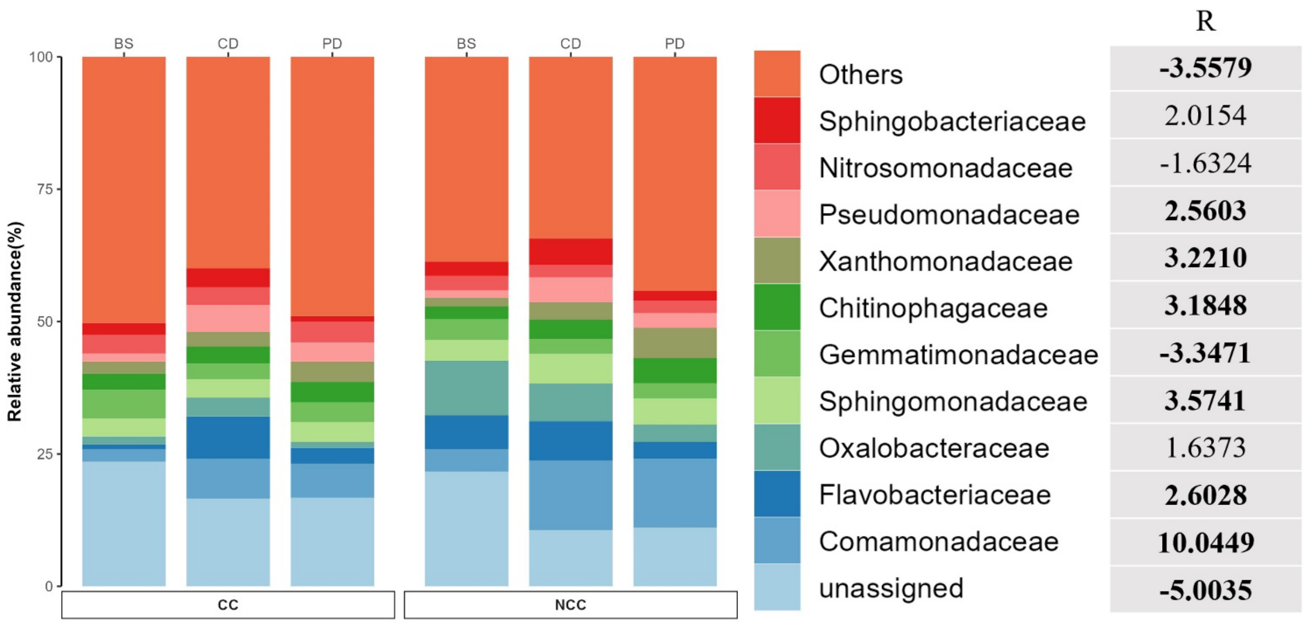

Supplementary Figure 2. Top 10 relative abundances of bacteria at the family level. The numbers on the right represent the R values of Spearman's correlation between the relative abundance of each

family and the abundance of morels in the soil. Significant correlation coefficients are noted in bold font ($P<0.05$).

1.2 Supplementary Tables

Supplementary Table 1. The value of soil physiochemical variables in every treatment

Type	pH	Organic matter(g/kg)	Total N (%)	Alkali-hydro N (mg/kg)	Total P (%)	Available P (mg/kg)	Total P(%)
BSNCC	7.61±0.0510	8.71±0.2988	0.06±0	42.8675±2.5526	0.0875±0.005	23.9725±1.1374	2.17±0.0408
CDNCC	7.82±0.0589	10.3375±0.9708	0.0725±0.005	42.0675±4.0302	0.1±0	45.9775±1.9705	2.1375±0.1034
PDNCC	8.3025±0.0741	11.6275±1.0761	0.085±0.0058	56.4675±2.6411	0.0975±0.005	50.1525±3.7679	2.0175±0.1144
BSCC	8.16±0.0698	21.8075±0.6841	0.14±0.0082	95.7675±5.1045	0.0975±0.005	56.17±3.7840	2.02±0.0990
CDCC	8.22±0.0490	23.7725±1.2854	0.1575±0.0171	105.2325±6.2291	0.1025±0.005	79.46±4.9381	2.02±0.0309
PDCC	7.9875±0.1223	23.8125±2.5197	0.17±0.0163	112.1675±8.8243	0.105±0.0058	80.3775±6.8255	1.9467±0.0189

Supplementary Table 2. Number of high-quality sequences in each replicate

Sample Name	Bactetial high-quality sequences
BSN1A	69,516
BSN1B	60,543
BSN1C	61,885
BSN1D	62,991
BSO1A	61,121
BSO1B	61,917
BSO1C	65,379
BSO1D	69,909
CDN1A	67,210
CDN1B	60,500
CDN1C	66,991
CDN1D	60,670
CDO1A	69,582
CDO1B	69,972
CDO1C	61,983
CDO1D	69,715
PDN1A	66,957
PDN1B	60,848
PDN1C	67,897
PDN1D	61,530
PDO1A	68,187
PDO1B	66,289
PDO1C	60,186
PDO1D	66,248
Sum	1,558,026
Mean	64,918
Median	65813.5
Max	69,972
Min	60,186

Supplementary Table 3. The value of alpha diversity in every treatment

Sample	Chao1	Phylogenetic_diversity
BSNCC	3549.50±299.18	203.73±15.83
CDNCC	2887.25±301.79	171.26±14.31
PDNCC	2857.75±270.60	174.97±14.96
BSCC	3262.00±135.43	203.52±15.52
CDCC	3200.50±378.46	192.71±16.41
PDCC	3191.50±304.61	194.51±11.06

Supplementary Table 4. Abundant indicator OTUs

name	s.BSNCC	s.BSCC	s.CDNCC	s.CDCC	s.PDNCC	s.PDCC	Phylum	Class	Order	Family	Genus
OTU_1	1	0	1	0	0	0	Proteobacteria	Gammaproteobacteria	Burkholderiales	Oxalobacteraceae	Massilia
OTU_5	0	0	0	0	1	1	Proteobacteria	Gammaproteobacteria	Cellvibrionales	Cellvibrionaceae	Cellvibrio
OTU_34	0	0	1	0	1	0	Proteobacteria	Gammaproteobacteria	Cellvibrionales	Cellvibrionaceae	Cellvibrio
OTU_7473	0	0	1	1	1	0	Proteobacteria	Betaproteobacteria	Burkholderiales	Comamonadaceae	unassigned
OTU_9	0	0	1	0	1	0	Proteobacteria	Betaproteobacteria	Burkholderiales	Comamonadaceae	Polaromonas
OTU_12	0	0	1	0	0	0	Proteobacteria	Betaproteobacteria	Burkholderiales	Comamonadaceae	Ramlibacter
OTU_7958	0	0	1	0	1	0	Proteobacteria	Betaproteobacteria	Burkholderiales	Comamonadaceae	unassigned
OTU_68	0	0	1	0	1	0	Proteobacteria	Betaproteobacteria	Burkholderiales	Comamonadaceae	unassigned
OTU_1107	0	0	1	0	1	0	Proteobacteria	Betaproteobacteria	Burkholderiales	Comamonadaceae	unassigned
OTU_22	0	0	0	0	1	1	Proteobacteria	Betaproteobacteria	Burkholderiales	Methylophilaceae	Methylophilus
OTU_17	0	0	1	1	1	1	Proteobacteria	Alphaproteobacteria	Rhizobiales	Rhizobiaceae	Agrobacterium
OTU_48	0	0	1	0	1	0	Proteobacteria	Alphaproteobacteria	Rhizobiales	Rhizobiaceae	Pararhizobium
OTU_21	1	0	1	0	1	0	Proteobacteria	Alphaproteobacteria	Sphingomonadales	Sphingomonadaceae	Sphingomonas
OTU_7	1	0	0	0	0	0	Actinobacteria	Actinobacteria	Micrococcales	Micrococcaceae	Pseudarthrobacter

OTU_69	0	1	0	0	0	1	Gemmatimonadota	Gemmatimonadetes	Gemmatimonadales	Gemmatimonadaceae	unassigned
OTU_235	1	0	1	0	1	0	Gemmatimonadota	Gemmatimonadetes	Gemmatimonadales	Gemmatimonadaceae	unassigned
OTU_133	1	0	1	0	0	0	Gemmatimonadota	Gemmatimonadetes	Gemmatimonadales	Gemmatimonadaceae	Gemmatimonas
OTU_58	0	1	0	0	0	0	Acidobacteriota	Acidobacteriae	Gp2	unassigned	unassigned
OTU_3045	0	1	0	1	0	1	Acidobacteriota	Acidobacteria_Gp6	Gp6	unassigned	unassigned
OTU_78	1	0	0	0	0	0	Acidobacteriota	Acidobacteria_Gp4	Gp4	unassigned	unassigned
OTU_387	1	0	0	0	1	1	Proteobacteria	Gammaproteobacteria	Xanthomonadales	Xanthomonadaceae	Lysobacter
OTU_29	0	0	0	0	1	0	Proteobacteria	Gammaproteobacteria	Xanthomonadales	Xanthomonadaceae	Stenotrophomonas
OTU_770	0	0	1	0	1	0	Bacteroidota	Bacteroidia	Sphingobacteriales	Sphingobacteriaceae	Daejeonella
OTU_85	1	0	1	0	0	0	Bacteroidota	Sphingobacteriia	Sphingobacteriales	Sphingobacteriaceae	Daejeonella
OTU_192	1	0	0	0	0	0	Firmicutes	Bacilli	Bacillales	Bacillaceae	Neobacillus
OTU_415	0	0	0	0	1	0	Bacteroidota	Chitinophagia	Chitinophagales	Chitinophagaceae	Lacibacter
OTU_64	1	0	0	0	0	0	Nitrospirota	Nitrospira	Nitrospirales	Nitrospiraceae	Nitrospira
OTU_6679	0	0	1	0	1	0	Proteobacteria	Alphaproteobacteria	Rhizobiales	Devosiaceae	unassigned
OTU_45	0	0	1	0	1	0	Proteobacteria	Alphaproteobacteria	Rhodobacterales	Rhodobacteraceae	unassigned
OTU_2781	0	0	1	0	0	0	Bacteroidota	Flavobacteriia	Flavobacteriales	Flavobacteriaceae	Flavobacterium
OTU_55	1	1	0	0	0	0	unassigned	unassigned	unassigned	unassigned	unassigned

OTU_9 110	1	0	0	0	0	0	unassigned	unassigned	unassigned	unassigned	unassigned

Supplementary Table 5. Spearman's correlations between abundant taxa and soil morel relative abundance

Name	t value	P value
Acidobacteriota	-3.257563066	0.003607046
Actinobacteria	-0.008157257	0.993565042
Alphaproteobacteria	7.418138105	2.02E-07
Bacteroidota	6.330306178	2.27E-06
Betaproteobacteria	7.874208809	7.67E-08
Firmicutes	-3.228247857	0.003865781
Gammaproteobacteria	2.470945459	0.021698354
Gemmatimonadota	-0.442446655	0.662485386
Nitrospirota	-0.654789662	0.519391149
unassigned	-2.948279636	0.007432235
